# The inflammatory/fibrotic axis across organs: myelofibrosis as a model of reversibility

**DOI:** 10.1172/jci.insight.202062

**Published:** 2026-07-08

**Authors:** Lucas Greven, Stijn N.R. Fuchs, Hélène F.E. Gleitz, Rebekka K. Schneider

**Affiliations:** 1 Institute for Cell and Tumor Biology, RWTH Aachen University Hospital, Aachen, Germany.; 2 Department of Developmental Biology and; 3 Oncode Institute, Erasmus University Medical Center, Rotterdam, Netherlands.

## Abstract

Fibrosis affects almost all organ systems, resulting in a dysfunctional extracellular matrix that impairs function and can lead to failure. Crosstalk between immune cells and the stromal environment exacerbates fibrosis in all organs and is an attractive therapeutic target. Here, we discuss recent findings regarding the cellular and molecular mechanisms that underlie inflammation and fibrosis across organs. We focus on how reciprocal immune/stromal signaling maintains fibrotic niches, outline strategies for therapeutic intervention beyond current antifibrotic agents, and highlight the bone marrow fibrotic disease myelofibrosis as a model for understanding, and ultimately reversing, fibrosis in human disease.

## The common language of fibrosis across organs

Fibrosis is characterized by persistent fibroblast activation, resulting in excess extracellular matrix (ECM) deposition, and is the common endpoint of chronic injury across organs. Fibrosis accounts for a large proportion of global disease burden, contributing to roughly 45% of all deaths in developed nations. Excess ECM stiffens tissues, impairs organ function, and ultimately leads to organ failure. Resolution of the wound-healing program is normally mediated through apoptosis of activated myofibroblasts; however, inflammatory cues — delivered by neutrophils, macrophages, and other immune cells — can persist, subsequently further activating fibroblasts into myofibroblasts that continue to secrete ECM. This dysregulation or “wound healing gone awry” concept can be provoked by diverse insults (infection, toxins, metabolic stress, malignancy, or genetic mutations) and be perpetuated by cytokines such as TGF-β, PDGF, and IL-1β. While altered wound healing presents as organ-specific manifestations, such as cirrhosis, glomerulosclerosis, and interstitial lung disease, the mechanisms of fibrosis across organs appear to be similarly reflected by an inflammatory/fibrotic cascade (1–3).

Fibrosis is traditionally viewed as irreversible scarring, but recent studies demonstrate that fibrotic tissues are dynamic and, under certain conditions, reversible. In lung fibrosis, for example, lipofibroblasts (LIFs) are programmed into activated myofibroblasts (aMYFs) during injury; however, pharmacologic agents have been shown to reverse this transition, promoting aMYF-to-LIF conversion and leading to fibrosis resolution (4). This paradigm shift is supported by the observation that abnormal ECM, which becomes the scaffold of a persistently fibrotic niche, can undergo remodeling during successful repair. Unfortunately, the success of therapeutic strategies that promote ECM remodeling remains confined largely to idiopathic pulmonary fibrosis (IPF), where two antifibrotic drugs, nintedanib and pirfenidone, are approved for use in patients. However, these drugs only slow down functional decline rather than reversing established fibrosis and show limited benefit when repurposed for dermal or systemic sclerosis–associated fibrosis. Thus, in general, no disease-modifying, antifibrotic therapies are currently available for fibrosis in most other organs, underscoring the need for new conceptual frameworks and treatments.

**Figure 1:**
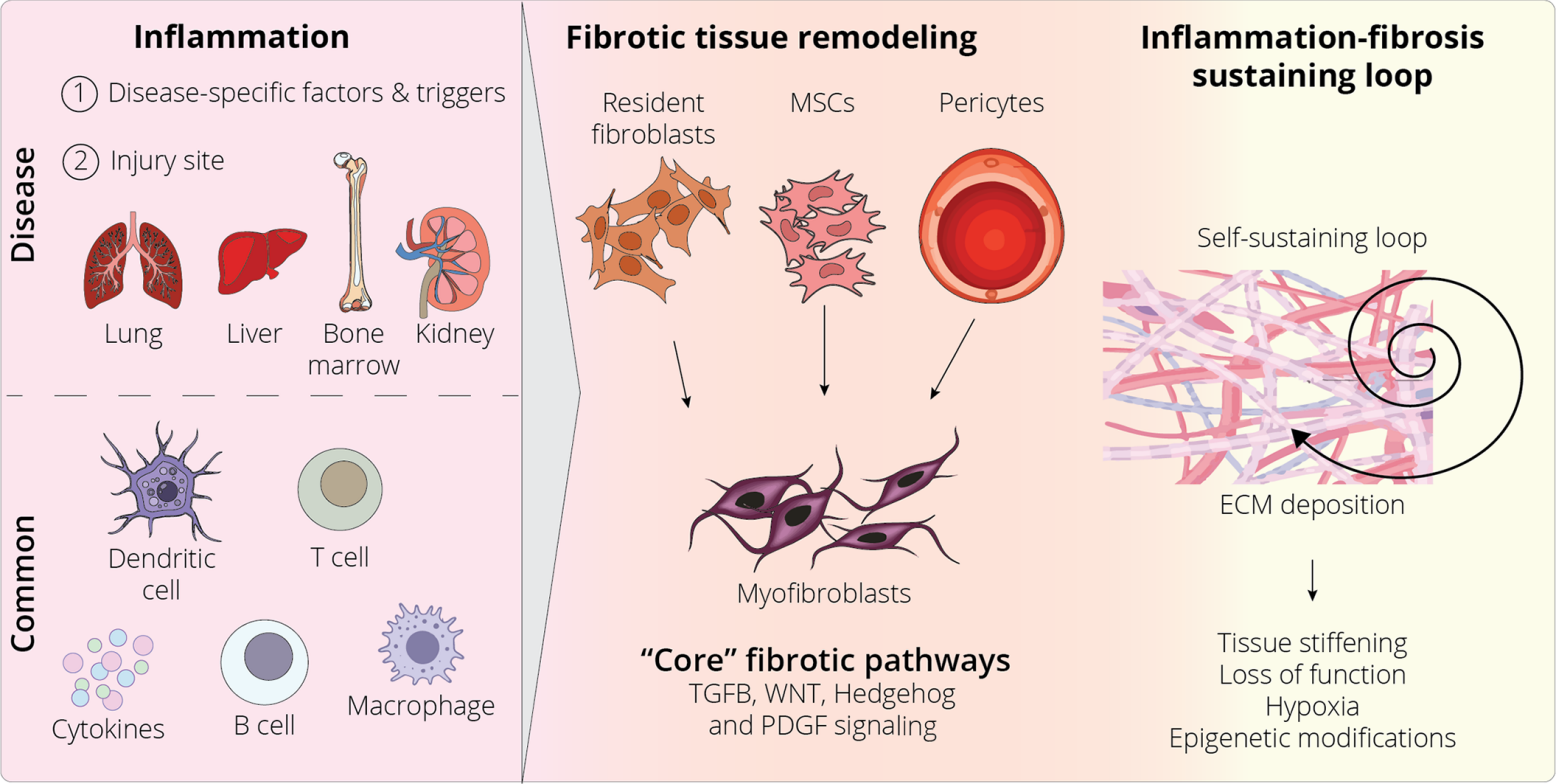
Graphical overview of the inflammation/fibrosis cycle across organs.

A unifying theme in fibrosis across organs is the dynamic interplay between stromal cells and the immune system (5, 6). Macrophages are recruited to sites of injury and secrete cytokines, including TGF-β, PDGF, and IL-1β, that induce fibroblast activation and differentiation. Activated fibroblasts subsequently produce chemokines, cytokines, and ECM components that modulate immune cell recruitment and polarization. Multiple signaling pathways, including TGF-β/SMAD, ERK, PI3K/AKT, and Wnt/Notch, integrate these inputs and govern the transition between repair and fibrosis. Together, these observations support a conceptual model in which fibrosis emerges from a dynamic inflammatory/stromal circuit. Tissue injury triggers innate immune activation and recruitment of monocytes, which differentiate into macrophage subsets that instruct stromal progenitors to adopt myofibroblast phenotypes. The resulting ECM remodeling further reinforces immune activation through mechanical and biochemical feedback signals. Importantly, this inflammatory/fibrotic circuit appears to be conserved across organs, suggesting that common cellular programs govern both the initiation and potential reversibility of fibrosis (Figure 1). Understanding how these circuits are disrupted or restored in specific disease contexts therefore provides a framework for therapeutic intervention (2, 7).

Myelofibrosis (MF), a fibrotic bone marrow (BM) disease, exemplifies this inflammatory/fibrotic loop (8). In MF, a malignant hematopoietic clone drives chronic cytokine release and aberrant signaling among megakaryocytes, macrophages, and mesenchymal stromal cells (MSCs), creating a TGF-β–rich environment that induces ECM deposition and stromal activation. Genetic fate-mapping has shown that perivascular Gli1^+^ MSCs migrate from the endosteal and vascular niches to become fibrosis-driving myofibroblasts. Moreover, ablation or pharmacologic inhibition of Gli1^+^ MSCs markedly reduces fibrosis. Single-cell mapping further reveals that distinct leptin receptor–positive (Lepr^+^) stromal subsets adopt profibrotic phenotypes, while the alarmin complex S100A8/A9, which is upregulated in mutant JAK2V617F hematopoietic cells, marks progression to overt MF. Inhibition of the S100A8/A9 axis with tasquinimod alleviates disease in preclinical models (9). Additionally, basophils and mast cells establish a TNF-rich signaling hub that reprograms stromal progenitors, with galectin-1 emerging as a biomarker and therapeutic target (10). Importantly, allogeneic hematopoietic stem cell transplantation (allo-HSCT) provides the first and only clinical evidence that BM fibrosis can be reversed in humans. Successful allo-HSCT not only eradicates the malignant clone but also restores a nonfibrotic stromal niche and normal hematopoiesis, offering a proof of concept that fibrosis, once established, remains a reversible and dynamic process when both the inflammatory driver and the fibrotic niche are reset.

## Macrophage–stromal crosstalk as a driver of fibrosis

### Initiation: injury, DAMPs, and monocyte recruitment.

**Figure 2:**
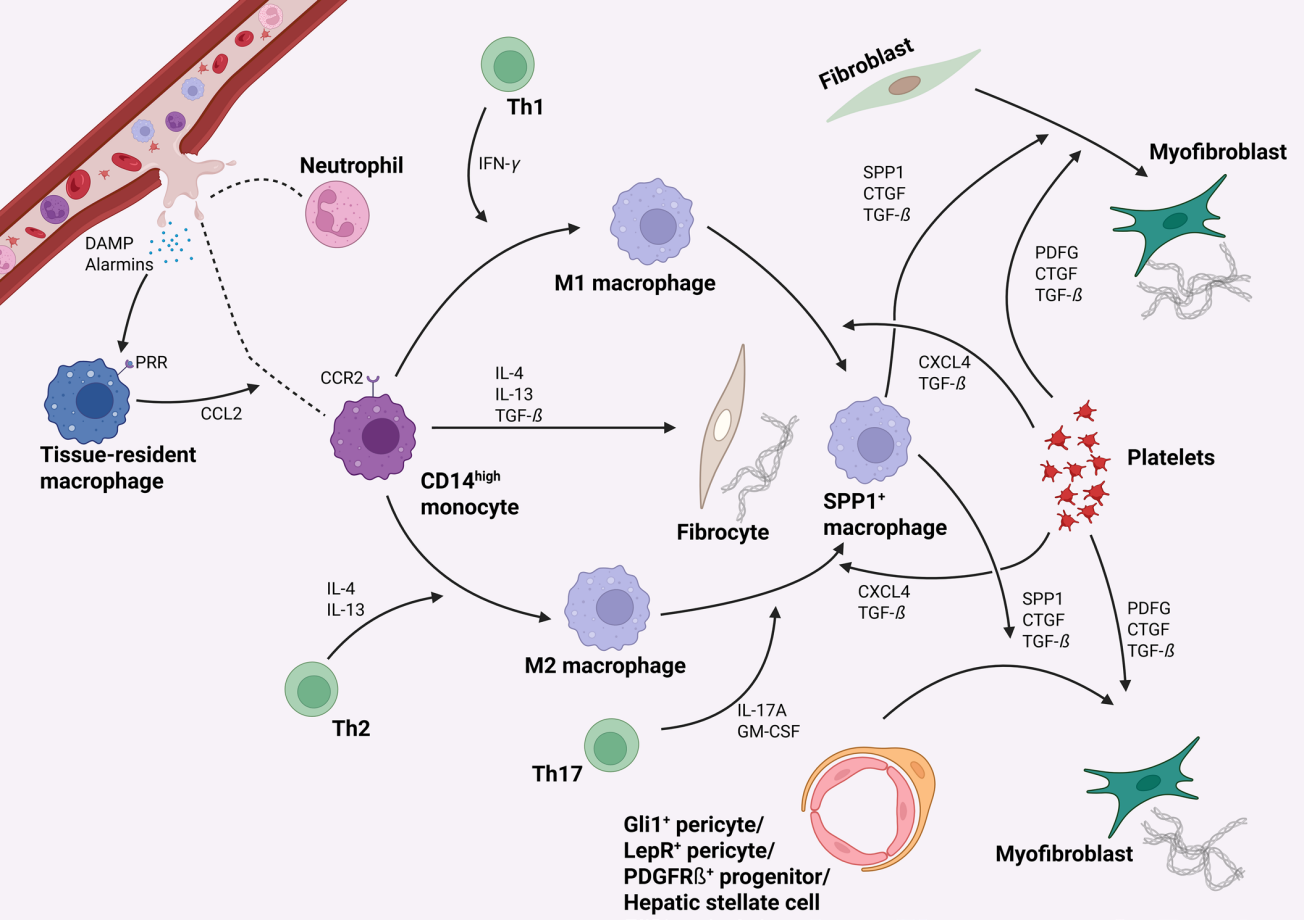
Immune cell–stromal interactions in solid organs drive fibrotic remodeling through both direct cell–cell communication and the secretion of soluble mediators, including cytokines and growth factors. Tissue injury or inflammation releases damage-associated molecular patterns (DAMPs) and alarmins that are recognized by tissue-resident macrophages via pattern recognition receptors (PRRs), leading to recruitment of circulating monocytes through the CCL2/CCR2 chemokine axis. Recruited Ly6C^hi^ (mouse) or CD14^hi^ (human) monocytes differentiate into monocyte-derived macrophages that polarize toward M1 or M2 states in response to local cues, including CD4^+^ Th cell cytokines, such as Th1-derived IFN-γ or Th2-derived IL-4 and IL-13. Under profibrotic conditions, Th17-derived interleukin-17A (IL-17A), granulocyte–macrophage colony-stimulating factor (GM-CSF), and megakaryocyte/platelet-derived CXC chemokine ligand 4 (CXCL4), together with transforming growth factor-β (TGF-β), drive differentiation of monocytes/macrophages into secreted phosphoprotein–positive (SPP1^+^) macrophages. These profibrotic SPP1^+^ macrophages, along with platelets, secrete mediators such as SPP1, connective tissue growth factor (CTGF), PDGF, and TGF-β, which activate fibroblasts and perivascular stromal cells, including glioma-associated oncogene homolog 1–positive (Gli1^+^) pericytes, leptin receptor–positive (LepR^+^) pericytes, PDGF receptor-β–positive (PDGFRβ^+^) progenitors, and hepatic stellate cells, depending on the tissue context. These cells differentiate into ECM-producing myofibroblasts that drive fibrotic remodeling. In addition, monocyte-derived fibrocytes also contribute to ECM deposition through IL-4–, IL-13–, and TGF-β–dependent signaling pathways.

Unresolved injury and persistent inflammation release damage-associated molecular patterns (DAMPs) and alarmins that activate pattern recognition receptors on resident macrophages and endothelium (Figure 2). This activation triggers cytokine/chemokine production and recruits Ly6C^hi^ (human CD14^hi^) monocytes via CCR2–CCL2 to sites of damage, where they differentiate into monocyte-derived macrophages with profibrotic programs. Depletion of these recruited macrophages alleviates experimental lung fibrosis, reducing collagen and alternative-activation markers (11, 12).

### Macrophage activation states: beyond the M1/M2 dichotomy.

Infiltrating monocytes were traditionally framed as M1 (pro-inflammatory) or M2 (repair/regulatory). In fibrosis, macrophage activation is highly plastic and context dependent. For example, subsets of M2-like macrophages (M2a/b/c/d) arise in response to distinct cues and can exert a variety of functions, including tissue repair, regulatory, antiinflammatory/profibrotic, or tumor-promoting activities (13–15). Thus, it has become clear that the simple M1/M2 paradigm is insufficient to capture the diversity of macrophage populations in fibrotic disease.

### Scar-associated macrophages.

Single-cell and spatial profiling have identified a conserved population of scar-associated macrophages (SAMs) across organs that are enriched for ECM-remodeling genes and high SPP1 (osteopontin) expression (16). In the liver, analogous TREM2^+^CD9^+^SPP1^+^ macrophages activate stellate cells, and similar populations appear in lung, heart, and kidney fibrosis (17–19). IL-17A, GM-CSF, and platelet-derived CXCL4 promote differentiation toward the SPP1^+^ state (17). Functionally, SPP1^+^ macrophages secrete osteopontin, TGF-β, and PDGFs, thereby fostering fibroblast proliferation and collagen deposition via SPP1-, FN1-, and SEMA3-mediated macrophage–stromal crosstalk (17). Two transcriptional SAM states have been described so far. SPP1^+^ macrophages that do not express marker-associated macrophage (MAM) markers appear to be enriched with age, while SPP1^+^MAM^+^ macrophages associate with matrix remodeling and predominate during active fibrosis (16).

## Adaptive immune modulation of fibrogenic macrophages

Th2 cytokines (IL-4/IL-13) drive alternative macrophage activation via IL-4Rα/STAT6-mediated induction of TGF-β1, which promotes fibrosis and contributes to later remodeling (20–22). Th17 cells sustain neutrophilic inflammation and expand SPP1^+^ macrophages through IL-17A/GM-CSF (18, 23). In contrast, Th1-derived IFN-γ induces SMAD7, suppresses TGF-β signaling, and is antifibrotic. In lung injury models, Th1 depletion exacerbates fibrosis, while Th17 loss mitigates fibrosis (24, 25). Tregs further complicate fibrosis outcomes, as Treg-derived amphiregulin stimulates fibroblasts, and Treg-specific amphiregulin loss attenuates liver fibrosis in metabolic dysfunction–associated steatohepatitis models (26).

## Stromal cell contributions and organ-specific context

Across organs, stromal cells, such as perivascular mesenchymal progenitors, fibroblasts, and pericyte-derived populations, are described to be the dominant source of myofibroblasts (27, 28). Lineage tracing studies have shown that Gli1^+^, FOXD1-lineage, ADAM12^+^, and Lepr^+^ stromal cells proliferate and differentiate into contractile, ECM-producing myofibroblasts after injury in kidney, liver, lung, heart, skin, and BM (27, 29–35). Myofibroblasts are generated in all organs; however, the source of these cells can be organ dependent. In the liver, α-SMA^+^COL1A1^+^ myofibroblasts are derived from quiescent hepatic stellate cells (36, 37). Kidney FOXD1^+^PDGFRβ^+^ stromal cells undergo STAT3-dependent migration and myofibroblast conversion (38). In the skin and muscle, ADAM12^+^ perivascular progenitors acquire α-SMA expression and secrete ECM following injury (35). In the BM, heterogeneous Lepr^+^ MSC subsets (ALPL^+^CXCL12^+^ adipogenic stromal cells and SPP1^+^WIF1^+^IBSP^+^SP7^+^BGLAP^+^ osteogenic stromal cells) expand and drive fibrosis. Gli1^+^ stromal progenitors fall within the BM compartment, with an osteolineage focus, and respond to alarmin and platelet-derived CXCL4/PF4 signaling (9, 34, 39, 40). Additionally, hematopoietically derived CD45^+^COL1A1^+^ fibrocytes, which arise from monocytes in response to IL-4/IL-13/TGF-β signaling, deposit collagen, and secrete TGF-β, are enriched in IPF lungs and JAK2V617F MF (41, 42).

Beyond fibroblast activation, macrophages also interact extensively with epithelial and endothelial cells, shaping tissue remodeling and fibrosis progression (7). In epithelial tissues such as the lung, liver, and kidney, injured epithelial cells release alarmins and cytokines that recruit and polarize macrophages, while macrophage-derived mediators — including TGF-β, IL-1β, and PDGF — promote epithelial dysfunction, epithelial–mesenchymal signaling, and barrier disruption. In parallel, endothelial cells contribute to the inflammatory–fibrotic niche by producing chemokines that facilitate monocyte recruitment and by undergoing endothelial-to-mesenchymal transition (EndMT), thereby generating additional ECM-producing cells (43, 44). Macrophage-derived cytokines and growth factors can directly promote EndMT and vascular remodeling, linking immune activation to structural alterations of the vascular niche. These interactions illustrate that fibrosis arises not only from fibroblast activation but also from coordinated communication among immune, stromal, epithelial, and vascular compartments.

## Innate sensing and inflammatory axes that prime fibrosis

DAMPs and alarmins, including S100A8/A9 HMGB1, engage TLRs to drive cytokine output, often through CCR2–CCL2–dependent immune cell recruitment. In BM fibrosis, S100A8/A9 is upregulated in stromal progenitors, marks disease progression, and when inhibited ameliorates murine MF (9). In MF, circulating HMGB1 and S100A8/A9 correlate with inflammatory activity (45). Across organs, TLR4 activation amplifies fibrosis, while TLR4 deficiency can attenuate fibrotic responses (46, 47). TLRs prime the NLRP3 inflammasome, subsequently activating caspase-1 and IL-1β to reinforce inflammatory–fibrotic loops, and pharmacologic NLRP3 inhibition reduces experimental liver fibrosis in mice.

## Cytokine effector landscape acting on macrophages and stroma

While the previous section describes how adaptive immune signals shape macrophage states, the following section focuses on the downstream cytokine networks that directly activate stromal cells and sustain ECM production. A complex cytokine network orchestrates the reciprocal activation of macrophages and stromal cells during fibrosis. Among the most potent mediators are IL-1α and IL-1β, which act as central amplifiers of inflammation and drivers of tissue remodeling. In experimental lung injury, IL-1β cooperates with bleomycin to induce pulmonary fibrosis in an IL-17A–dependent manner (23). In the BM, selective deletion of IL-1β in JAK2V617F mutant hematopoietic cells markedly ameliorates fibrosis, highlighting a pathogenic role for IL-1β in inflammatory niche remodeling (48). IL-6, particularly through its trans-signaling pathway, activates STAT3 in fibroblasts, thereby promoting the expression of ECM–associated genes and enhancing fibroblast proliferation (49). These results establish IL-6 as a critical link between chronic inflammation and sustained matrix production. Similarly, TNF-α bridges immune activation and fibrogenesis by upregulating TGF-β1 expression in fibroblasts, thereby integrating inflammatory and canonical profibrotic pathways. Type 2 cytokines, notably IL-4 and IL-13, further shape both macrophage polarization and fibroblast activation. Acting through the IL-13R–IL-13α2–IL-4Rα receptor complex, IL-13 induces TGF-β1 expression and stimulates myofibroblast differentiation. Myeloid IL-4Rα signaling supports activation of alternative macrophages that promote tissue repair and fibrosis progression but also participate in later phases of resolution (20, 21). Together, these cytokine-mediated pathways establish a tightly interwoven network that sustains inflammation, drives stromal activation, and reinforces the chronicity of fibrotic disease.

HMGB1 is a prototypical DAMP that is elevated across hematologic malignancies and fibrotic disorders (50–52). The redox state of HMGB1 dictates its function, with reduced HMGB1 being chemotactic and disulfide HMGB1 acting as a pro-inflammatory cytokine (53). Fibroblasts migrate directionally toward secreted HMGB1 (54), and HMGB1 can induce expression of inflammatory cytokines (IL-1β, TNF-α, IL-6, IL-18) (55). HMGB1 expression, at both the mRNA and protein level, is significantly elevated in BM fibrosis and also associated with systemic sclerosis, cystic fibrosis, and liver fibrosis (55–58).

Fibrosis emerges from a feed-forward loop in which DAMP-driven monocyte recruitment and context-specific macrophage states (notably SPP1^+^ SAMs) instruct and amplify stromal myofibroblast programs via a TLR/inflammasome/cytokine axis, with organ-specific stromal progenitors and fibrocytes executing sustained ECM deposition. Together, these observations illustrate that macrophage–stromal interactions form a central regulatory node of fibrogenesis. Consequently, many emerging antifibrotic strategies aim to interrupt these inflammatory–stromal circuits, by targeting macrophage activation states, neutralizing DAMP signaling, or eliminating fibrosis-driving stromal populations.

## Therapeutic modulation of fibrosis: from cytokines to matrix remodeling

As outlined above, a wide array of mediators orchestrates activation and persistence of myofibroblasts during fibrosis. Therapeutic efforts have therefore focused on interfering with key cytokine and growth factor pathways that sustain chronic inflammation and excessive ECM deposition. Among these, the IL-1 and IL-6 axes have received considerable attention as central inflammatory drivers linking immune activation to fibrotic remodeling.

IL-1–directed therapies have shown promise in both experimental and clinical settings. The IL-1 receptor antagonist anakinra has demonstrated antifibrotic potential in preclinical models of pulmonary, renal, and arthrofibrosis, primarily by suppressing IL-1–driven inflammatory signaling (59–61). Similarly, the IL-1β–neutralizing mAb canakinumab attenuates systemic inflammation and hematopoietic skewing (62). In the CANTOS trial, which enrolled more than 10,000 patients with cardiovascular risk, canakinumab treatment suppressed hepcidin, myeloid activation, and inflammatory cytokines (63). Strikingly, the strongest responses were observed in patients harboring clonal hematopoiesis mutations, such as TET2 or DNMT3A, highlighting the role of IL-1β in driving inflammation-resistant clonal expansion and ineffective erythropoiesis (64).

IL-6 is implicated in fibrotic remodeling across organs, including the lung, liver, heart, and BM (65–68). Elevated IL-6 levels correlate with disease severity, anemia, and splenomegaly in myeloproliferative neoplasms (MPNs) (69–72). In vitro studies indicate that IL-6 stimulation of MSCs induces myofibroblast-like features, including α-SMA upregulation (73, 74). mAbs that target IL-6 (siltuximab) or its receptor (tocilizumab) have therefore emerged as potential immunotherapies in chronic inflammatory and neoplastic diseases with fibrotic components (75–77).

Beyond individual cytokines, combined blockade of the IL-1 family signaling complex represents a broader antiinflammatory approach to treating fibrosis. The IL-1 receptor accessory protein (IL1RAP) serves as a coreceptor for IL-1R, IL-33R, and IL-36R and is highly upregulated in systemic sclerosis. Targeting IL1RAP with a humanized LALA-mutated IgG1κ antibody (CAN10) inhibited IL-1α/β, IL-33, and IL-36 signaling and reduced collagen deposition and myofibroblast activation in dermal and lung fibrosis models. Moreover, CAN10 may hold promise for hematologic fibrotic disorders such as MF (75, 78). A first-in-human clinical trial using this antibody is currently ongoing in plaque psoriasis (79). While these cytokine-targeted strategies demonstrate tangible benefits, systemic immune modulation carries inherent risks, such as infection and/or off-target suppression, emphasizing the need for organ-specific or locally delivered interventions.

As discussed above, DAMP-driven inflammation reinforces fibrosis by perpetuating macrophage activation and stromal priming; therefore, therapeutic strategies to neutralize innate danger signals are of interest. The S100A8/A9 alarmin complex is an attractive target, as it is markedly upregulated in murine and human MPN–associated BM fibrosis. Moreover, S100A8/A9 alarmin is typically absent in stromal cells, with expression shifting from mutant hematopoietic clones to fibrosis-driving mesenchymal cells during disease progression. Pharmacological inhibition of S100A8/A9 binding with tasquinimod ameliorated cytopenia and markedly reduced BM fibrosis in JAK2V617F models (9, 39, 80). HMGB1-neutralizing Abs have demonstrated efficacy in models of diabetic cardiomyopathy, cystic fibrosis, and arthritis, while the orally available HMGB1 inhibitor SB17170, converted to its active form SB1703, is under phase II evaluation for IPF (81). While the context-dependent function of the HMGB1 redox state complicates pharmacologic targeting, accumulating evidence positions this prototypical DAMP as a promising target that connects inflammation, stromal activation, and clonal hematopoiesis.

TGF-β remains the central profibrotic master regulator. It is produced by multiple cell types, including megakaryocytes, macrophages, platelets, endothelial cells, and stromal cells, and orchestrates myofibroblast differentiation and ECM deposition. TGF-β inhibition has therefore been explored at several mechanistic levels and as potential therapeutic targets. Activation of latent TGF-β, which is secreted in complex with latency-associated peptide, can be prevented by targeting its activators. Inhibition of the matrix protein thrombospondin-1 or α_v_β_3_/α_v_β_5_ integrins suppresses TGF-β1/SMAD3 signaling, collagen production, and fibrosis in peritoneal, renal, and cardiac injury models (82–86). Direct neutralization strategies have included the pan–TGF-β antibody fresolimumab (GC1008), which reduced both myofibroblast markers and TGF-β–inducible genes in patients with systemic sclerosis (87). However, systemic TGF-β blockade causes adverse effects, including cardiovascular damage and impaired wound healing in preclinical models (88). More selective approaches, such as the ligand trap AVID200 targeting TGF-β1 and TGF-β3, showed encouraging results, including improved BM morphology and hematologic parameters without cardiac or dermatologic toxicity, in a phase Ib clinical trial in patients with MF (NCT03895112) (89). Downstream inhibition of TGF-β signaling via ALK5/TGF-βR1 kinase blockade (galunisertib) has been primarily assessed in oncology, but preclinical models of liver, kidney, and lung fibrosis demonstrate substantial antifibrotic potential (90–95). Similarly, neutralization of CTGF with pamrevlumab has proven effective in preclinical cardiac and peritoneal fibrosis and has shown safety and disease-modifying activity in patients with IPF (96–98).

Despite progress in approaches to modulate inflammatory and growth factor pathways, these strategies often fail to fully reverse established fibrosis because they do not eliminate the effector cells responsible for ECM deposition. Consequently, attention has shifted toward direct targeting of fibrosis-driving cells. Elegant preclinical work has demonstrated the feasibility of selective fibroblast depletion using chimeric antigen receptor (CAR) T cell therapy. In cardiac injury models, fibroblast activation protein–specific (FAP-specific) CAR T cells removed activated fibroblasts, reduced interstitial collagen, and improved cardiac function (99). A lipid nanoparticle mRNA delivery platform then enabled transient in vivo generation of FAP-CAR T cells that achieved similar antifibrotic effects without ex vivo manipulation (100). More recently, CAR T cells targeting PDGFRβ^+^ fibroblasts, which are abundant in chronic kidney disease, effectively reduced fibrosis in mouse models and human kidney organoids, outperforming FAP-CAR T cells (101). Approaches that eradicate or reprogram ECM-producing fibroblasts may enable sustained tissue recovery where cytokine inhibition alone fails.

Finally, the fibrotic matrix itself has emerged as a therapeutic target. Enzymes of the lysyl-oxidase (LOX) and LOX-like (LOXL1–4) families catalyze covalent cross-linking of collagens and elastins, stabilizing the stiff ECM that is characteristic of chronic fibrosis. In preclinical models, Ab-mediated inhibition of LOXL2 with AB0023 attenuated fibrosis across organs (102–104); however, the humanized analog simtuzumab failed in phase II trials for patients with liver fibrosis and MF (105). In contrast, small-molecule LOXL2 inhibitors, such as GB2064, have shown favorable safety, target engagement, and preliminary improvements in BM fibrosis, while the pan-LOX inhibitor PXS-5505 demonstrated good tolerability and early evidence of reduced marrow collagen in patients with MF (106). Broader inhibition of the LOX family, particularly LOXL4, appears more effective than selective LOXL2 blockade, producing stronger suppression of collagen cross-linking and fibrosis resolution in preclinical lung and liver models (107, 108). Together, these results underscore ECM remodeling enzymes as tractable targets for restoring tissue elasticity and interrupting the mechanical feed-forward loop that sustains fibrosis.

Across all therapeutic classes, systemic toxicity and limited tissue penetration remain major challenges. Emerging strategies are increasingly focused on tissue-selective delivery, including Ab drug conjugates, nanoparticles, or prodrugs that preferentially accumulate in fibrotic niches. Together, these multifaceted approaches to attenuate inflammatory cytokines, block DAMP-driven amplification, modulate TGF-β and CTGF pathways, deplete activated fibroblasts, and reduce ECM cross-linking converge on a paradigm for true fibrosis reversal that combines immune modulation, stromal targeting, and matrix remodeling into an integrated therapeutic framework.

## BM fibrosis and MF as a paradigm for fibrosis reversal

Among all fibrotic diseases, BM fibrosis in MF provides a rare clinical example where complete structural reversal of fibrosis can occur. In MF, allo-HSCT promotes regression, even with advanced reticulin and collagen fibrosis, that includes restoration of normal marrow architecture and reappearance of adipocytes and hematopoietic niches. Thus, the BM provides a unique and powerful model for true fibrosis regression in humans. Mechanistically, reversal likely reflects a combination of effects: elimination of the inflammatory malignant clone, repopulation by donor-derived hematopoietic and immune cells that suppress profibrotic signaling, and gradual reprogramming or replacement of fibrosis-driving stromal cells. Despite this curative potential, allo-HSCT remains a high-risk and aggressive therapy that is associated with considerable morbidity and mortality. Only a subset of younger and fit patients qualifies for transplantation. A better understanding of the cellular and molecular mechanisms that enable fibrosis reversal in this context will be crucial to replicate this regenerative process through targeted and less toxic interventions in the future.

MF represents the most aggressive form of the Philadelphia chromosome–negative MPN, which also encompasses polycythemia vera and essential thrombocythemia (31, 109). These clonal stem cell disorders arise from somatic mutations in JAK2, CALR, or MPL, all converging on constitutive activation of the JAK/STAT pathway. This signaling promotes myeloproliferation and excessive cytokine release, fueling a chronic inflammatory milieu rich in IL-6, TNF-α, IL-1β, and TGF-β. In the BM, these cytokines activate MSCs and fibroblasts, leading to pathological ECM deposition and reticulin fibrosis. Over time, marrow failure develops, accompanied by cytopenia and compensatory extramedullary hematopoiesis in the spleen. Thus, inflammation and fibrosis are intertwined hallmarks of disease progression, representing both cause and consequence of the disrupted hematopoietic niche.

## Profibrotic stromal and hematopoietic crosstalk

In MF, fibrosis primarily originates from mesenchymal stromal progenitors, including Lepr^+^, Gli1^+^, and nestin^+^ perivascular cells (9, 34, 110), which transdifferentiate into α-SMA^+^ myofibroblasts under cytokine stimulation or promote a neuropathy-like state in the BM. α-SMA^+^ myofibroblasts deposit collagens, fibronectin, and fibrillar ECM proteins that alter marrow stiffness and impede normal hematopoietic stem cell function. Megakaryocytes serve as central amplifiers of this process by secreting TGF-β, PDGF, and CXCL4/PF4, which drive stromal activation and recruit profibrotic macrophages (39, 111). During disease progression, the marrow microenvironment undergoes a shift from inflammatory to fibrotic signaling, dominated by TGF-β/SMAD, SPP1, and S100A8/A9 pathways, which consolidate a self-sustaining profibrotic feedback loop between the hematopoietic clone and the stromal niche.

Beyond the canonical stromal progenitors, other niche compartments also have been described to contribute to fibrosis. EndMT enables endothelial cells to lose vascular characteristics and acquire mesenchymal, ECM-producing phenotypes, disrupting vascular integrity and amplifying fibrosis (112). Dysregulation of differentiation of cells in the osteolineage and osteosclerosis accompany advanced stages of MF, reflecting abnormal coupling between bone formation and fibrosis. Importantly, alarmins such as S100A8/A9 act as key molecular mediators linking the malignant clone to its fibrotic niche. These proteins are upregulated in mutant hematopoietic cells and are transferred to stromal progenitors, where they activate TLR4 and RAGE signaling, sustaining inflammation, ECM production, and myofibroblast persistence.

## Therapeutic landscape and niche-directed interventions

The JAK1/2 inhibitor ruxolitinib remains the standard of care for MF, effectively reducing splenomegaly and inflammatory cytokine burden while improving constitutional symptoms. However, ruxolitinib exerts minimal effect on fibrosis regression, clonal allele burden, or overall survival, and its use is limited by cytopenia and resistance (113, 114). Consequently, current therapeutic strategies increasingly aim to target the fibrotic BM niche and its pathogenic signaling loops (Table 1).

One of the most advanced antifibrotic candidates is PRM-151 (zinpentraxin alfa), a recombinant form of human pentraxin-2 that modulates macrophage differentiation and inhibits myofibroblast activation. In phase II trials, patients with MF were treated with PRM-151 alone or in combination with ruxolitinib, and clinical benefits included a 37% mean spleen size reduction, 54% improvement in symptom score, and measurable regression of MF fibrosis in one-third of patients (115). While PRM-151 is no longer in development for MF, it remains under evaluation for other organ fibroses and underscores the potential of macrophage-modulating antifibrotic therapies. TGF-β signaling is another promising therapeutic target. The selective TGF-β1/3 ligand trap AVID200 inhibits ligand–receptor interaction without affecting the homeostatic TGF-β2 isoform. In an investigator-led phase Ib study in patients with advanced MF, AVID200 treatment reduced plasma TGF-β levels, decreased SMAD2 phosphorylation and stromal proliferation, and resulted in more than 50% symptom reduction in nearly half of patients (89). Clinical improvement was observed particularly after extended treatment cycles, suggesting a time-dependent remodeling effect on the fibrotic niche (116). Additional strategies under exploration include mAbs that directly target TGF-β (e.g., fresolimumab) or small molecules, such as galunisertib, that inhibit the TGF-β receptor I kinase (ALK5), which has shown antifibrotic efficacy in preclinical models.

In parallel, luspatercept, a recombinant fusion protein comprising the activin receptor IIB domain linked to human IgG1 Fc, acts as a ligand trap for activins and GDF11, thereby inhibiting downstream SMAD2/3 signaling and promoting erythroid differentiation. In clinical studies, luspatercept improved anemia in MF, achieving transfusion independence in up to one-third of transfusion-dependent patients receiving concurrent ruxolitinib. It is currently being evaluated in a phase III placebo-controlled trial (117).

Pharmacologic inhibition of Gli1, a key effector of the hedgehog signaling pathway that is expressed by myofibroblast progenitors, represents another avenue for targeting stromal activation. In preclinical MPN models, the small-molecule Gli1 inhibitor GANT61 prevented myofibroblast differentiation and ameliorated BM fibrosis, highlighting a potential strategy to block the stromal activation cascade at its origin (27, 29, 118).

Finally, the identification of S100A8/A9 as a central hematopoietic/stromal signaling axis has led to translational efforts targeting this alarmin complex. The small molecule tasquinimod, which prevents S100A8/A9 binding to its receptors TLR4 and RAGE, markedly reduced fibrosis and improved hematopoiesis in preclinical MPN models (9, 80). Based on these findings, two clinical trials are underway to test tasquinimod as monotherapy or in combination with ruxolitinib, aiming to directly disrupt the inflammatory cross-talk that drives fibrosis progression (119, 120).

## From transplantation to regeneration

Allo-HSCT in BM fibrosis provides one of the most compelling clinical proofs of principle that even advanced fibrotic architecture can be reversed in humans. In patients with MF, successful transplantation leads to the elimination of the malignant hematopoietic clone and initiates a cascade of microenvironmental changes that progressively restore tissue homeostasis. Early after transplantation, inflammatory cytokine levels decline and macrophage activation states normalize, reflecting the rapid disruption of the chronic inflammatory circuits that sustain fibrosis. This normalization is followed by gradual remodeling of the ECM, accompanied by the disappearance of reticulin and collagen fibers that previously dominated the marrow architecture. Over time, donor-derived hematopoietic and immune cells repopulate the marrow niche and reestablish balanced immune signaling. As these regulatory networks are restored, fibrosis-driving stromal populations lose their pathological activation state.

These clinical observations fundamentally challenge the long-standing view of fibrosis as a permanent scar and instead support the concept that fibrosis is a dynamic and actively maintained tissue state that depends on continuous signaling between inflammatory, immune, and stromal compartments. In this context, the reversal of BM fibrosis following transplantation can be understood as a so-called “natural experiment” in human regenerative biology: once the initiating driver is removed and inflammatory circuits are reset, the tissue retains a remarkable capacity to remodel itself and regain functional architecture. This regenerative remodeling involves coordinated changes across multiple cellular layers of the niche, including hematopoietic cells, immune regulators, stromal progenitors, and ECM-producing fibroblasts.

**Figure 3:**
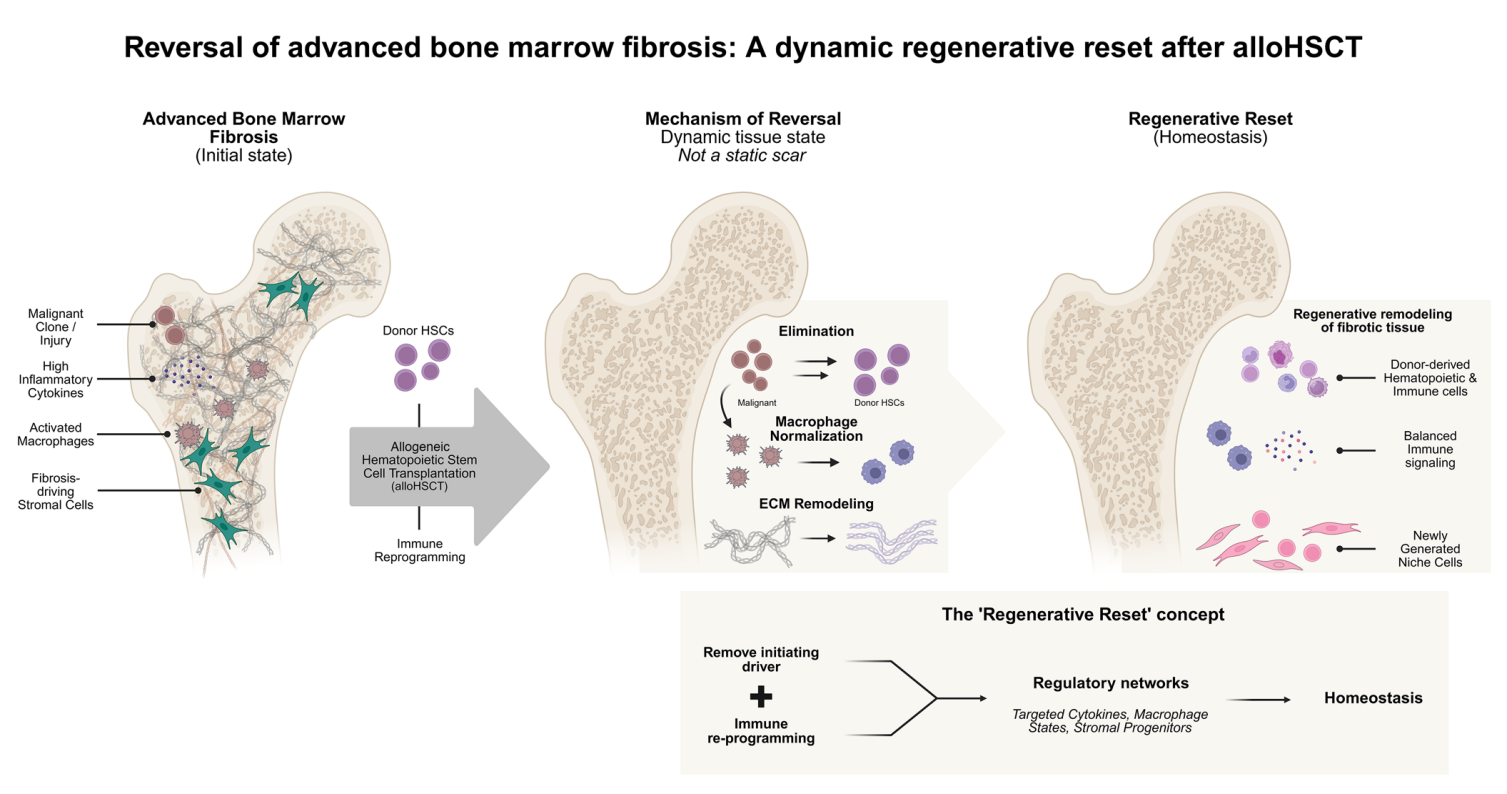
The regenerative reset of BM fibrosis following allo-HSCT. The pathologically altered bone marrow (BM) niche in BM fibrosis is induced by injury in the form of a malignant hematopoietic clone causing high concentrations of inflammatory cytokines that activate macrophages (and megakaryocytes) into a profibrotic state. This induces the fibrotic transformation of stromal cells and ECM deposition over time. Following allogeneic hematopoietic stem cell transplantation (allo-HSCT), a sequential transition occurs: the elimination of the malignant clone (the injury stimulus) leads to a rapid decline in inflammatory signaling, which triggers the normalization of macrophages toward a homeostatic state and facilitates the enzymatic remodeling of the fibrotic matrix. This “regenerative reset” culminates in a restored niche where donor-derived hematopoietic and immune cells maintain balanced signaling, allowing stromal cells to revert to a quiescent phenotype or be replaced by healthy niche cells. This natural experiment demonstrates that removing the initiating driver and reprogramming the immune microenvironment can drive the transition from advanced fibrosis back toward tissue homeostasis.

The success of allo-HSCT therefore demonstrates that organ fibrosis can normalize when the pathogenic drivers that sustain it are extinguished and stromal homeostasis is reestablished (Figure 3). The critical challenge now is to translate the biological lessons learned from this clinical scenario into therapeutic strategies that reproduce key aspects of the regenerative reset without requiring ablative transplantation. Achieving this goal requires a detailed understanding of how donor hematopoiesis restores immune equilibrium, dampens DAMP signaling, and interrupts the self-reinforcing inflammatory/stromal circuits that maintain fibrosis. Equally important is deciphering how activated stromal populations, including fibrosis-driving fibroblasts and mesenchymal progenitors, either revert to quiescence or are replaced during tissue remodeling. Future studies combining lineage tracing, spatial transcriptomics, and longitudinal marrow biopsies will be critical to determine whether fibrosis-driving stromal cells revert to quiescence, undergo apoptosis, or are replaced by newly generated niche cells during this regenerative process.

Importantly, the mechanistic insights gained from BM fibrosis are likely to extend beyond hematologic disease. Across multiple organs — including liver, lung, kidney, and heart — fibrosis emerges from similar feedback loops linking immune activation, macrophage polarization, stromal progenitor activation, and ECM deposition. The BM therefore provides a uniquely informative system in which the full cycle of fibrosis initiation, maintenance, and reversal can be studied in humans. Unlike most solid organs, the BM is readily accessible for longitudinal sampling and high-resolution histologic analysis, allowing researchers to track dynamic changes in cellular composition, inflammatory signaling, and matrix remodeling over time. As such, BM represents a powerful in vivo model system, a living laboratory, for understanding how fibrotic tissues transition back toward homeostasis.

By dissecting the cellular and molecular programs that enable fibrosis regression after allo-HSCT, the field may uncover broadly applicable strategies to therapeutically reprogram fibrotic tissues. Such strategies could include targeted modulation of inflammatory drivers, manipulation of macrophage states, elimination or reprogramming of fibrosis-driving stromal cells, and stimulation of regenerative niche formation. Ultimately, insights derived from this uniquely accessible regenerative process may help guide the development of molecularly targeted therapies capable of inducing true fibrosis reversal across organs.

## Conclusions and outlook

Across organs, fibrosis is increasingly recognized not as a terminal scarring endpoint but as a dynamic and potentially reversible process. The BM provides a particularly powerful paradigm for this concept. In MF, mutant hematopoietic and progenitor cells orchestrate a chronic inflammatory environment that activates Gli1^+^ and Lepr^+^ stromal progenitors to transdifferentiate into ECM-producing myofibroblasts. When these profibrotic and inflammatory signals are dampened — whether through JAK inhibition, TGF-β blockade, alarmin targeting, or hematopoietic replacement after allo-HSCT — stromal cells can gradually revert toward a homeostatic, hematopoiesis-supporting phenotype. These observations demonstrate that BM fibrosis is not fixed but plastic and that its resolution can be therapeutically induced. Because of its well-characterized cellular composition, accessibility, and regenerative capacity, the BM represents an exceptional model system for studying the principles of fibrosis reversal. Lessons from this tissue illuminate a broader truth: across organs, the interplay between chronic inflammation, immune–stromal crosstalk, and mechanical remodeling drives fibrogenesis, and interventions that restore this balance can re-enable endogenous repair.

Looking ahead, the integration of single-cell, spatial, and temporal omics technologies will fundamentally reshape our understanding of how inflammatory, stromal, and vascular compartments interact to sustain or resolve fibrosis. In MF and beyond, these approaches will allow precise mapping of lineage trajectories, signaling networks, and spatial cell–cell interactions that determine the fate of fibrotic lesions. Such insights will guide the development of microenvironment-directed therapies that can be combined with oncogene- or mutation-targeted treatments, tackling both the clonal driver and its supportive niche. The next generation of antifibrotic therapies will likely move beyond symptom control to achieve true disease modification — restoring organ architecture, function, and regenerative capacity. MF thus serves not only as a clinical challenge but also as an experimental blueprint for how reversing the inflammatory and stromal circuits that sustain fibrosis could ultimately enable organ regeneration across systems.

## Conflict of interest

RKS is a cofounder and shareholder of Sequantrix GmbH and has received a research grant from Active Biotech.

## Funding support

- HG by a Gilead Research Scholar Award in Oncology/Hematology, a ZonMW VENI grant, an Erasmus Medical Center Fellowship, a Dutch Cancer Society (KWF) Exploration grant, and an NWO ENW M1 grant.
- RKS by European Research Council (ERC) grants (Rewind-MF ERC-CoG 101124542; deFIBER ERC-StG 757339 and PoC DeAlarmin) and a ZonMW VIDI grant.
- Grants of the Deutsche Forschungsgemeinschaft (DFG) (German Research Foundation) to HG (417911533) and RKS (504777725; 417911533; 514007497).
- RKS as member of the E:MED Consortia Fibromap and the consortium CureFib by the German Ministry of Education and Science (BMBF).
- LG as part of the Research Training Group (RTG 2375) by the DFG.

## Figures and Tables

**Table 1:**
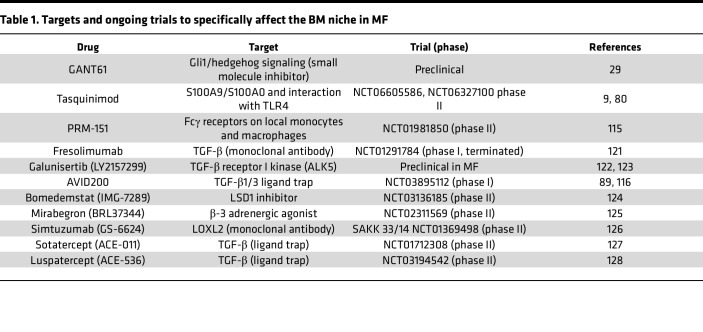
Targets and ongoing trials to specifically affect the BM niche in MF

## Version 1 07/08/2026

Electronic publication
